# Obstructive sleep apnea and pulmonary function in patients with severe obesity before and after bariatric surgery: a randomized clinical trial

**DOI:** 10.1186/2049-6958-9-43

**Published:** 2014-08-09

**Authors:** Isabella C Aguiar, Wilson R Freitas, Israel R Santos, Nadua Apostolico, Sergio R Nacif, Jéssica Julioti Urbano, Nina Teixeira Fonsêca, Fabio Rodrigues Thuler, Elias Jirjoss Ilias, Paulo Kassab, Fernando SS LeitãoFilho, Rafael M Laurino Neto, Carlos A Malheiros, Giuseppe Insalaco, Claudio F Donner, Luis VF Oliveira

**Affiliations:** 1 Sleep Laboratory; Master’s and PhD Degree Pos Graduation Programs in Rehabilitation Sciences, Nove de Julho University (UNINOVE), Rua Vergueiro 235, Liberdade CEP 01504–001, Sao Paulo, SP, Brazil; 2 Surgery Department, Santa Casa de Misericórdia Hospital, Sao Paulo, SP, Brazil; 3 Medicine School, Universidade de Fortaleza, Fortaleza, CE, Brazil; 4 Bariatric Surgery Service, Conjunto Hospitalar do Mandaqui, Sao Paulo, SP, Brazil; 5 A. Monroy Institute of Biomedicine and Molecular Immunology, National Research Council of Italy, Palermo, Italy; 6 Mondo Medico, Multidisciplinary and Rehabilitation Outpatient Clinic, Borgomanero, NO, Italy

**Keywords:** Bariatric surgery, Pulmonary function, Severe obesity, Sleep disorders, Ventilatory muscles

## Abstract

**Background:**

The increasing prevalence of obesity in both developed and developing countries is one of the most serious public health problems and has led to a global epidemic. Obesity is one of the greatest risk factors of obstructive sleep apnea (OSA), which is found in 60 to 70% of obese patients mainly due to the buildup of fat tissue in the upper portion of the thorax and neck. The aim of the present randomized clinical trial is to assess daytime sleepiness, sleep architecture and pulmonary function in patients with severe obesity before and after bariatric surgery.

**Methods:**

This randomized, controlled trial, was designed, conducted, and reported in accordance with the standards of The CONSORT (Consolidated Standards of Reporting Trials) Statement. Patients were divided into a bariatric surgery group and control group. The clinical evaluation was performed at the Sleep Laboratory of the Nove de *Julho*University (Sao Paulo, Brazil) and consisted of the collection of clinical data, weight, height, body mass index (BMI), measurements of neck and abdomen circumferences, spirometry, maximum ventilatory pressure measurements, standard overnight polysomnography (PSG) and the administration of the Berlin Questionnaire and Epworth Sleepiness Scale.

**Results:**

Fifty-two patients participated in the present study and performed PSG. Out of these, 16 underwent bariatric surgery. After surgery, mean BMI decreased from 48.15 ± 8.58 to 36.91 ± 6.67 Kg/m^2^. Significant differences were found between the preoperative and postoperative periods regarding neck (p < 0.001) and waist circumference (p < 0.001), maximum inspiratory pressure (p = 0.002 and p = 0.004) and maximum expiratory pressure (p = 0.001 and p = 0.002) for women and men, respectively, as well as sleep stage N3 (p < 0.001), REM sleep *(*p *=* 0.049) and the apnea-hypopnea index (p *=* 0.008).

**Conclusions:**

Bariatric surgery effectively reduces neck and waist circumference, increases maximum ventilatory pressures, enhances sleep architecture and reduces respiratory sleep disorders, specifically obstructive sleep apnea, in patients with severe obesity.

**Trial registration:**

The protocol for this study was registered with the World Health Organization (Universal Trial Number: U1111-1121-8873) and Brazilian Registry of Clinical Trials – ReBEC (RBR-9k9hhv).

## Background

The increasing prevalence of obesity in both developed and developing countries is one of the most serious public health problems and has led to a global epidemic [1,2]. Epidemiological studies have shown that obesity is associated with comorbidities, such as cardiovascular disease [3,4], metabolic disease (e.g., diabetes) [5,6], chronic kidney disease and immunological disorders [7]. Obesity also exerts an important and complex influence on the respiratory system. Furthermore, excessive body weight may cause impairment in pulmonary function and can lead to a restrictive or, occasionally, an obstructive pulmonary disorder. The obese subject presents impairment in respiratory mechanics causing adverse effects on the pulmonary function such as increase in respiratory work and reduction of lung volumes. Several mechanisms have been suggested as possible effects of obesity on lung function [8].

Obesity is one of the greatest risk factors of obstructive sleep apnea (OSA), which is found in 60 to 70% of obese patients mainly due to the buildup of fat tissue in the upper portion of the thorax and neck. The mechanism by which obesity can favor the onset of OSA is not fully elucidated. Increased fat deposits in the neck region cause soft tissue enlargement and contribute to a critical narrowing of the airways. However, the upper airways are not always narrowed by these fat deposits, as evidenced by the fact that a substantial percentage of morbidly obese patients do not present OSA [8-10]. The incidence of respiratory sleep disorders in patients with morbid obesity is 12-to-30-fold higher than that in the general population [11].

The aim of a change in diet or surgical intervention in patients with obesity is addressed to improve both health and quality of life through sufficient weight loss in order to reduce or even eliminate comorbidities and promote psychological wellbeing [12]. Bariatric surgery is often the only effective treatment cure in severe obesity, which remains largely refractory to diet and pharmacologic treatment [13]. We hypothesize that in severely obese patients significant weight loss induced by bariatric surgery would provide an effective improvement in pulmonary function and sleep quality. Study endpoints were (i) to assess the effect of bariatric surgery on sleep parameters, mainly the apnea-hypopnea index as measured by standard overnight polysomnography (PSG) and (ii) symptoms of daytime sleepiness and risk of OSA.

The aim of the present study is to compare daytime sleepiness, respiratory sleep disorders, sleep architecture and pulmonary function variables in subjects with morbid obesity before and after bariatric surgery.

## Methods

**Figure 1:**
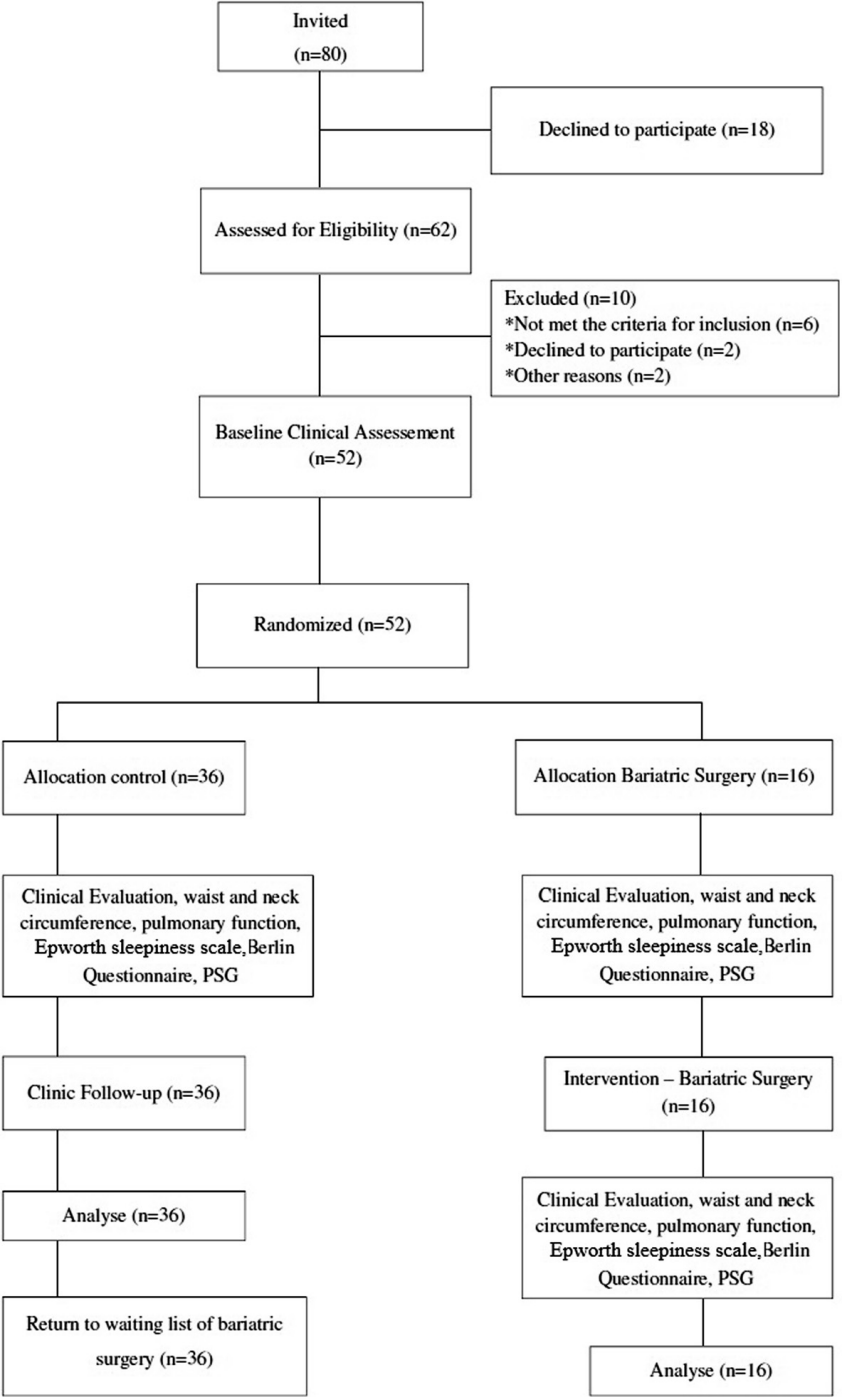
Flowchart representing the study design.

A controlled, prospective, randomized clinical trial was carried out based on our previously published study protocol [14], following approval from the Human Research Ethics Committee of the Universidade Nove de Julho(Brazil) under protocol number 220506/2009. This study is registered with the World Health Organization (Universal Trial Number: U1111-1121-8873) and the Brazilian Registry of Clinical Trials (RBR-9k9hhv). This randomized, controlled trial, was designed, conducted, and reported in accordance with the standards of The CONSORT (Consolidated Standards of Reporting Trials) Statement, which represents the gold standard in evaluating health care interventions [15]. Figure 1 displays the flow diagram describing the study design.

Eighty subjects with morbid obesity treated at the Gastric Surgery Service of the Santa Casa de Misericordia and at the Bariatric Surgery Group of the *Conjunto Hospitalar Mandaqui* and addressed to the Sleep Laboratory of the Nove de Julho University were recruited for participation in the study. All institutions are located in the city of Sao Paulo (Brazil). After being informed of the objectives and procedures, those who agreed to participate signed a statement of informed consent.

The inclusion criteria were morbid obesity (body mass index [BMI] between 40 and 50 kg/m^2^) or BMI between 35 and 39.9 kg/m^2^ with associated comorbidities. The exclusion criteria were: any active malignancy, active alcohol and/or drug abuse, dementia or treatment-refractory psychiatric diseases leading to an inability to provide informed consent, and use of medication that may interfere with the sleep structure, such as hypnotic drugs or stimulants of the central nervous system.

The patients were divided into a control group and a bariatric surgery group (2:1). Numbers were generated from a randomization table at a central office, where a set of sealed, opaque, sequentially numbered envelops was used to conceal the allocation. Each envelop contained a card stipulating in which group the individual would participate. The randomization process was adopted due to the very large number of patients to be submitted to bariatric surgery and the inability of the surgical service to meet all the demands. At the end of a mean period of 90 days, the patients in the control group returned to the waiting list to undergo bariatric surgery. All participants were clinically stable.

Polysomnography (PSG) was performed before and after bariatric surgery (gastric banding) in the bariatric surgery group with a 90-day interval between evaluations.

The clinical evaluation of the subjects, according to pre-specified primary and secondary outcome measures, was performed at the Sleep Laboratory of the Nove de Julho University (Sao Paulo, Brazil) and consisted of clinical data collection, that included heart and respiratory rates, peripheral blood pressure, weight, height, BMI, measurements of neck and abdomen circumference, spirometry, respiratory pressure measurements, PSG and the administration of the Berlin Questionnaire and Epworth Sleepiness Scale (ESS).

The anatomic references for waist and neck circumference measurements were standardized. Waist circumference was measured at the mid-point between the lower margin of the last rib and the iliac crest, whereas the neck circumference was measured horizontally at the level of the cricoid cartilage [14].

The pulmonary function test was performed with the patient seated comfortably using the KoKo PFT System spirometer (version 4.11) (nSpire Health, Inc; Louisville, CO, USA) following the guidelines for pulmonary function tests determined by the *SociedadeBrasileira* de Pneumologia e Tisiologia [16]. The measurement of maximal inspiratory and expiratory pressure [17] (MIP and MEP) generated by the respiratory muscles was performed using an aneroid manometer (Ger-Ar Com., Produtos Medicos Ltda., Sao Paulo, Brazil). We used the predicted values recommended by Neder et al. [18].

For the sleep study, type 1 PSG monitoring was performed using the 16-channel Embla sleep analysis system (A10 version 3.1.2 Flaga, Hs. Medical Devices, Reykjavík, Iceland), involving electroencephalogram, electromyogram, electrooculogram, nasal and oral airflow, thoracic and abdominal respiratory effort, digital pulse oximetry and electrocardiogram. A technician with experience in PSG monitored the patients. All signals were recorded continuously. Sleep stages were visually scored in 30-second epochs. Each PSG recording was analyzed manually, under blind conditions by the same examiner with experience in scoring PSG recordings based on the international standards established by the Academy of Sleep Medicine Manual for Scoring Sleep and Associated Events [19].

Apnea was scored when airflow ceased for 10 s or longer according to usual criteria. Hypopnea was recorded when the airflow was below 50% for at least ten seconds, followed by > 3% oxyhemoglobin desaturation. The apnea/hypopnea index (AHI) was calculated as the number of (apneas + hypopneas)/ hour of sleep time [19].

The ESS is a self-administered, eight-item scale that takes few minutes to respond [20]. These questions were developed based on identified low stimulus activities promoting sleepiness. Items address daily lifestyle activities and the subject is asked to rate their likelihood of dozing in each situation, from: “would never doze” (0) to “high chance of dozing” (3). The ESS provides a cumulative score between 0 and 24, with higher numbers indicating greater daytime sleepiness. Observing an order of magnitude above the mean as excessive sleepiness, we categorize ESS 0 to 8 as normal, 9 to 12 as mild, 13 to 16 as moderate, and greater than 16 as severe [21].

The Berlin Questionnaire (BQ) was used in this study. This clinical history questionnaire has recognized efficacy in distinguishing individuals at greater risk for OSA. BQ have ten items organized into three categories: snoring and apnea (5 items), daytime sleepiness (4 items) and systemic arterial hypertension and obesity (1 item). All marked responses are scored as positive. Two or more positive categories indicate high risk [22].

### Statistical analysis/sample size

A previous study published by Lettieri*et* al. [23] identified a mean reduction in the AHI of 23.4 events/hour in severe obese patients submitted to bariatric surgery (expected size effect). Using a standard deviation of 22.8 events/hour from the same study and considering α = 0.05 and power = 80%, the sample was estimated to be 15 patients [23].

When applicable, descriptive and numerical data were reported as mean ± SD. Comparisons between groups were performed using the Student’s *t* test or Mann–Whitney U test, depending on the type of distribution. For the comparison of variables within the same group, either the paired t-test or Wilcoxon test was used, as appropriate. All tests were 2 tailed and p minor than 0.05 was presumed to represent statistical significance. All analyses were performed using the SPSS version 19.0 (Chicago, IL, USA).

### Quality control

In order to ensure data quality, the physiotherapists and physicians in charge of the data acquisition in this study received specific training. Periodic external monitoring was performed to verify the adequate application of the methodology in collecting information and performing the different evaluations. The results of the pre and postoperative procedures were analyzed by blinded evaluators.

## Results

Between June 2011 and September 2013, eighty patients were recruited for the study, and eighteen subjects refused to participate and ten were excluded for not meeting the eligibility criteria. Therefore, our final sample consisted of 52 obese patients on the waiting list for bariatric surgery. After randomization, all these patients underwent evaluation protocol, including PSG. Sixteen patients (13 women) who composed the bariatric surgery group were evaluated before and after surgical intervention.

The demographic and anthropometric characteristics of all participants were showed in Table 1. We would like to draw attention to the values for BMI, waist circumference and neck that showed statistically significant differences after bariatric surgery.

**Table 1:** Demographic and anthropometric characteristics of sample

**Variables**	**Control (n = 36)**	**Pre Surgery (n = 16)**	**After Surgery (n = 16)**	**p**
				**(after vs. pre)**
Age (Years)	42.30 ± 11.87	40.08 ± 9.86	40.08 ± 9.86	
Height (cm)	167.00 ± 0.10	157.00 ± 10.00	157.00 ± 10.0	
Weight (Kg)	118.48 ± 23.13	118.92 ± 19.68	90.44 ± 10.53	ns
BMI (Kg/m^2^)	46.20 ± 6.13	48.15 ± 8.58	36.91 ± 6.67	0.004*
Waist circ. (cm)	126.77 ± 12.86	122.25 ± 10.40	103.50 ± 9.72	<0.001*
Neck circ. (cm)	41.96 ± 3.74	41.83 ± 3.65	36.20 ± 2.44	<0.001*

BMI, Body mass index; Neck circ, Neck circumference; Waist circ, Waist circumference.

*p derived by paired Student’s t test.

The values of pulmonary function, MIP and MEP before and after bariatric surgery can be seen in the Table 2. The spirometric test findings were expressed in absolute values and as percentage of predicted values, revealing the presence of obstructive and/or restrictive respiratory disorders. The spirometry was normal in 11 patients (68.75%) and showed restrictive lung function impairment in five patients (31.25%) from pre surgery group. There were no significant differences in lung function parameters between the control group and surgery group, whereas in the surgery group pre bariatric and after bariatric data showed significant differences.

**Table 2:** Pulmonary function and maximal ventilatory pressures before and after bariatric surgery

**Variables (n = 16)**	**Control (n = 36)**	**Pre surgery**	**After surgery (n = 16)**	**p**
				**(after vs. pre)**
FVC (L)	3,06 ± 0,47	2,96 ± 0,63	3,33 ± 0,80	0,002**
FVC (% of pred.)	102 ± 21,46	97,75 ± 18,65	110,50 ± 16,95	0,016**
FEV_1_ (L)	2,45 ± 0,34	2,38 ± 0,52	2,71 ± 0,72	0,003**
FEV_1_ (%)	97,57 ± 20,92	91,92 ± 21,24	107,00 ± 20,23	0,022**
FEV_1_/FVC (L)	0,79 ± 0,08	0,80 ± 0,07	0,81 ± 0,07	ns
FEV_1_/FVC (% of pred.)	79,43 ± 8,06	80,08 ± 7,28	81,50 ± 7,44	ns
MIP (cmH2O)				
Female	53,61 ± 17,63	52,67 ± 18,91	83,75 ± 10,78	0,002*
Male	62,31 ± 21,32	61,18 ± 23,11	87,54 ± 9,83	0,004*
MEP (cmH2O)				
Female	52,37 ± 15,69	53,58 ± 16,88	80,50 ± 13,31	0,001*
Male	62,31 ± 17,25	60,65 ± 18,36	84,96 ± 7,86	0,002*

FEV_1_, Forced expiratory volume in the first second; FVC, Forced vital capacity; MEP, Maximal expiratory pressure; MIP, Maximal inspiratory pressure; * p derived by paired Student’s t test; ** p derived by Wilcoxon Test.

**Figure 2:**
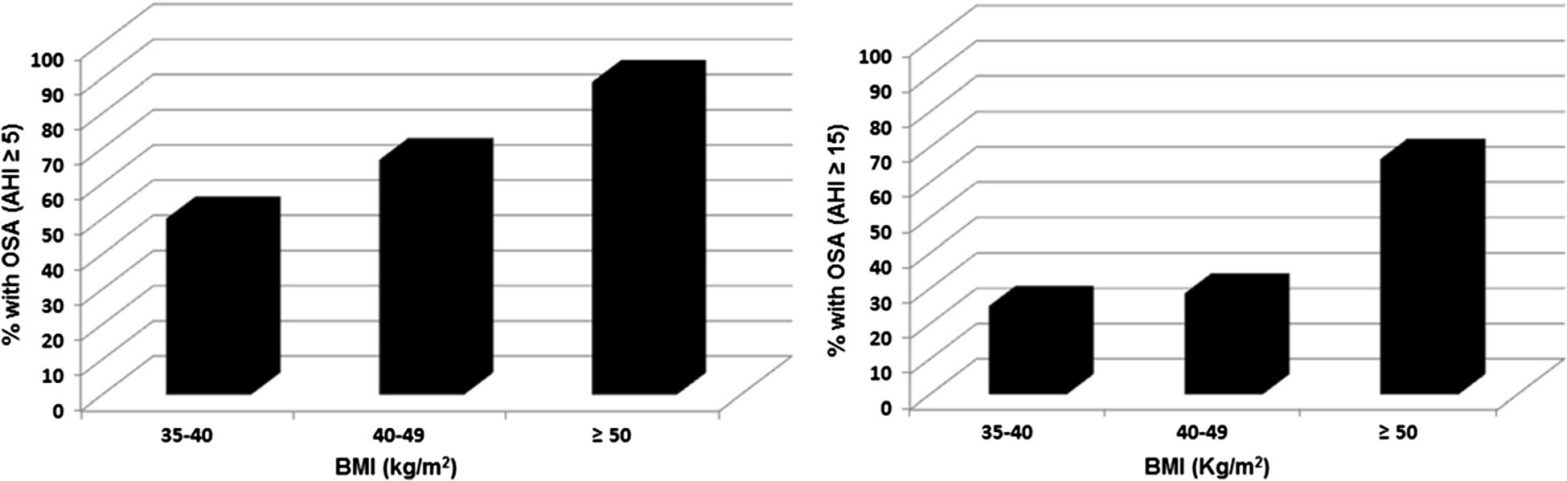
Percentage of patients with OSA across BMI strata, according to AHI.

**Figure 3:**
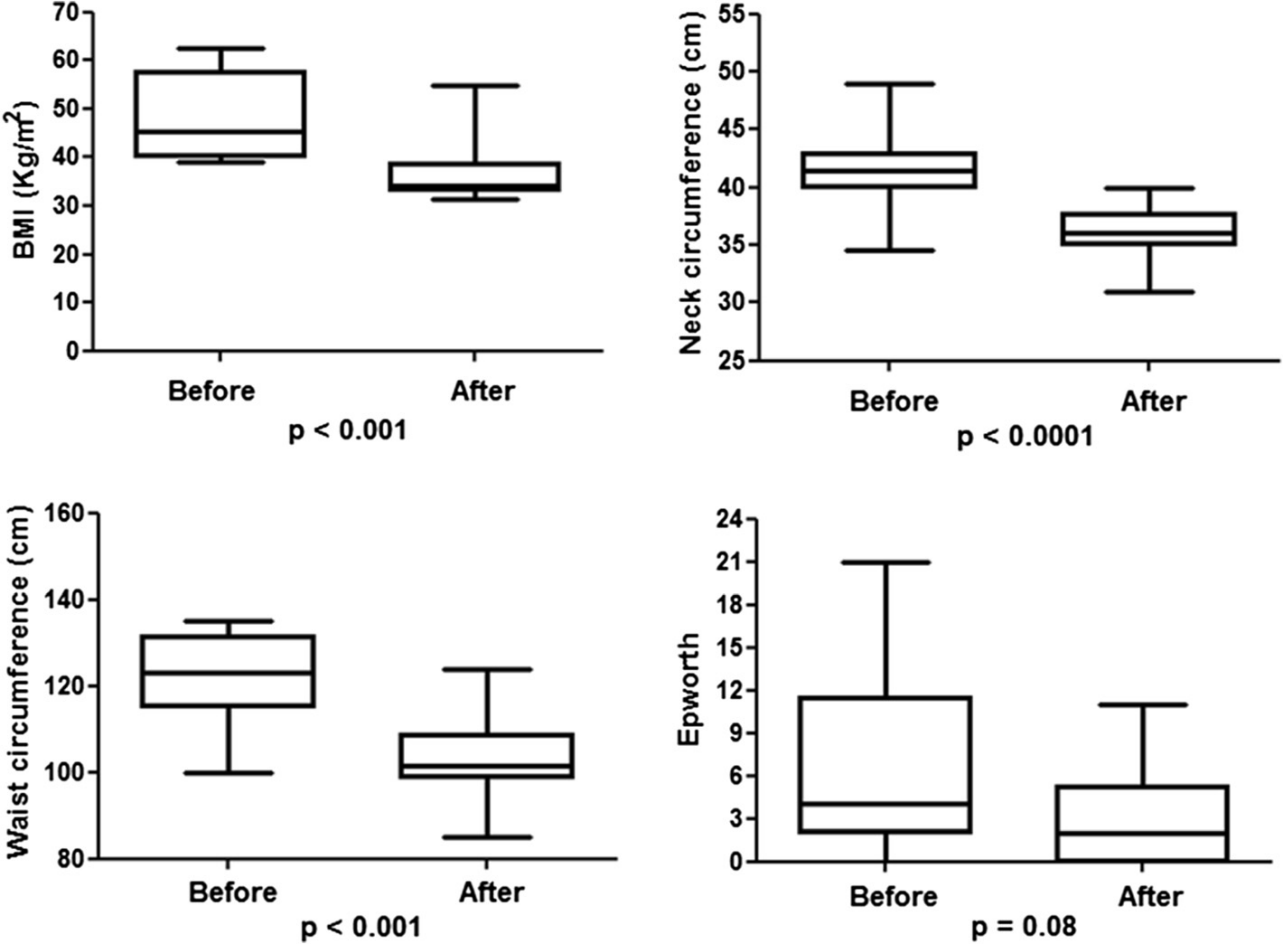
Distribution of BMI, neck circumference, waist circumference and ESS scores.

The sleep physiological variables analyzed, ESS and Berlin questionnaire of patients involved in this study are show in Table 3. The stratification of the BMI in the different degrees of AHI severity can be seen in Figure 2. A positive association was found between these variables, as approximately 85% of patients with BMI > 50 kg/m^2^ exhibited OSA. The mean values of BMI, neck and waist circumference and ESS scores can be seen in Figure 3. Significant differences were found between the preoperative and postoperative periods, except for the ESS. The control group (n = 36) did not undergo the second PSG in this trial due to the cost of the exam and the difficulty in the accessibility of the sleep laboratory as well as the fact that the individuals in this group did not demonstrate any changes in anthropometric variables at the follow-up while on the waiting list for surgery.

**Table 3:** Sleep physiological variables

**Variables**	**Control (n = 36)**	**Pre surgery (n = 16)**	**After surgery (n = 16)**	**p**
				**(after vs. pre)**
% SE	73.43 ± 9.16	74.27 ± 15.35	77.42 ± 23.73	ns
% 1NREM	10.80 ± 12.14	11.37 ± 10.78	14.34 ± 9.17	ns
% 2NREM	49.92 ± 18.71	57.52 ± 20.37	43.88 ± 5.80	ns
% 3NREM	15.68 ± 7.32	13.59 ± 10.62	17.54 ± 5.70	0.000*
% REM	23.60 ± 13.27	17.52 ± 11.91	24.24 ± 8.26	0.049*
Mean SaO2	92.66 (78 to 97)	93.30 (87 to 97)	94.3 (86.6 to 98)	ns
Nadir SaO2	79.90 (50 to 94)	83.25 (70 to 94)	85 (70 to 95)	ns
Wake SaO2	97 (94 to 98)	96.38 (95 to 99)	97 (94 to 99)	ns
Mean AHI	15.34 ± 9.14	15.65 ± 15.51	6.26 ± 7.57	0.008*
ESS	9.18 ± 5.34	6.92 ± 6.54	3.00 ± 3.56	ns
BQ	1.45 ± 0.50	1.75 ± 0.45	1.08 ± 0.28	0.001*
AHI (<5)	10	2	9	
AHI ( 5 < 15)	14	7	6	
AHI (15 < 30 )	5	4	1	
AHI (≥30)	7	3	0	

AHI, Apnea/hypopnea index; BQ, Berlin Questionnaire; ESS, Epworth Sleepiness Scale; NREM, No Rapid-eye-movement sleep; REM, Rapid-eye-movement sleep; SaO_2_, Arterial oxygen saturation; SE, Sleep Efficiency. * p derived by paired Student’s t test.

The patients undergone to bariatric surgery in the present study exhibited a significant reduction both of BMI (p = 0.004) and of waist circumference of 23.34% and 15.33% (p < 0.001), respectively, at three months following bariatric surgery. A significant reduction of 13.45% (p < 0.001) in neck circumference was found and it was positively correlated with reductions of body weight (r = 0.68, p = 0.015) and BMI (r = 0.58, p = 0.049). The PSG pre**-** surgery revealed an AHI of 15.65 ± 15.51, which was positively correlated with BMI (r = 0.57, p = 0.050). The results of PSG after bariatric surgery showed a significant reduction of AHI medium (6.26 ± 7.57). Before surgery, seven patients had a moderate to severe AHI, and after the intervention, only one patient showed a moderate score, as shown in Table 3.

The Berlin questionnaire revealed a significant improvement between the preoperative (1.75 ± 0.45) and postoperative (1.08 ± 0.28) periods (p = 0.001). This finding was in accordance with the gold standard for the identification of OSA (PSG), for which a significant decrease in the postoperative AHI (p = 0.008) was found.

## Discussion

The use of bariatric surgery for weight loss among individuals with morbid obesity has increased since the end of nineties of the last century. Bariatric surgery for individuals considered severely obese is reported to be associated with reductions in comorbidities, obesity-related complications and all-cause mortality [24]. Comparing surgical and conventional weight loss therapy for the management of OSA, Dixon et al. [25] demonstrated greater weight loss in the surgically treated group [25].

Waist circumference allows a practical evaluation of central adiposity, which is best correlated with visceral fat, and its association with BMI is an important marker for predicting the risk of systemic arterial hypertension, dyslipidemia and metabolic syndrome [26]. The patients in the present study exhibited a significant reduction of both BMI and waist circumference at three months following bariatric surgery. These findings are in agreement with those reported by Karakas et al*.*[27], who also describe significant reductions of these variables [27].

In a study involving obese patients, Stepien*et* al. [28] found a positive correlation between neck circumference and body weight [28]. Likewise, in the present study the significant reduction of neck circumference was positively correlated with the reduction of body weight. Mean neck circumference in the preoperative period was similar to the mean measurement reported in a study involving 296 women with morbid obesity indicated for bariatric surgery [29].

Due to its high prevalence rate, OSA is currently considered a public health problem and a clear association with obesity was observed [30,31]. Indeed, studies involving patients with morbid obesity submitted to bariatric surgery report that the subsequent weight loss is accompanied by a reduction in the severity of OSA [23,32]. Peppard *et* al*.*[33] found that a 10% increase in body weight implied a 32% increase in AHI and a 10% decrease in body weight led to a 26% decrease in AHI [33]. In the present study, preoperative PSG revealed an AHI positively correlated with BMI and the postoperative PSG revealed a 60% reduction of mean AHI.

In the present study, correlations between the AHI (stratified by severity) and obesity indices revealed a tendency toward a greater frequency of OSA with the increase in BMI. This finding is in agreement with data reported in a study by Ravesloot*et* al. [30], who found a high prevalence rate of OSA (69.9%) in a large sample of patients with morbid obesity (n = 289) on the waiting list for bariatric surgery, among whom 40% exhibited criteria for the diagnosis of severe OSA [30].

Severe obesity is associated with important changes in respiratory pattern, with consequences in lung function that have not been fully clarified yet. The literature reports a reduction of maximum pressure generated by the contraction of the ventilatory muscles due to the biomechanical disadvantage and an increase in respiratory work. The impairment in respiratory mechanics, characterized by a reduction of thoracic compliance due to the buildup of fat, a reduction of tidal volume and a consequent increase in respiratory rate, are positively correlated with the degree of obesity [34,35].

In a study involving obese individuals between 20 and 64 years of age, Magnani*et* al*.*[36] found that obesity did not affect maximum ventilatory pressures generated by the respiratory muscles, as no significant differences were found in the comparison with the reference values for normality proposed by Neder*et* al. [18]. The present data are in disagreement with these findings, as a significant reduction of maximum ventilatory pressures was found in the preoperative period in comparison to the reference values and a significant increase in pressures occurred in the postoperative period [18,36]. Similar results are reported in a study assessing physical and functional respiratory performance among obese Brazilian women, who exhibited considerably lower MIP and MEP in comparison to the Neder*et* al. [18] reference values [37].

The assessment of maximal ventilatory muscle strength is of considerable importance in individuals with severe obesity due to the possibility of cardiopulmonary complications stemming from the impairment of these muscles, especially in the postoperative period [38]. In a study by Soares*et* al*.*[37], obese subjects did not exhibit changes in the respiratory pattern or lung volume/capacity in comparison to predicted values. These findings are in disagreement with those reported in our preliminary study [34] and our recent findings as well as with results described by Wei et al. [32], who reported a significant change in forced vital capacity (FVC) and forced expiratory volume in the first second (FEV_1_) among individuals with severe obesity prior to bariatric surgery.

In the present sample, five patients exhibited a restrictive respiratory pattern and the others had values within the normal range when compared to the predicted values. Significant improvements of FVC (p= 0.002), FVC% (p = 0.016), FEV_1_ (p = 0.003) and FEV_1_% (p = 0.003) were found after bariatric surgery. These results are in agreement with those reported in a study carried out by Wei et al. [32].

A number of epidemiological studies report a strong association between severe obesity and sleep-related respiratory disorders, especially OSA, with a very high prevalence rate ranging from 50 to 89% [29,33,39,40]. OSA and daytime sleepiness are common manifestations in obese individuals and can lead to psychological and physiological problems, with a consequent negative effect on quality of life [39,41]. In the present study, the mean ESS score was 6.92 ± 6.54 prior to bariatric surgery and 3.00 ± 3.56 in the postoperative period. These findings are in agreement with those reported in studies carried out by Sharkey et al. [39] (involving 269 patients with morbid obesity) and Yeh *et* al. [41], who reported preoperative ESS scores of 6.3 ± 4.8 and 8.2 ± 4.7, respectively [39,41].

Anatomical and physical risk factors for OSA in the general population are well known. Using the Berlin questionnaire on a sample of 744 patients in primary care clinics, Netzer*et* al*.*[22] detected the presence of symptoms and identified risk factors for OSA [22]; the questionnaire demonstrated 86% sensitivity for the presence of respiratory disorders, which is fivefold more sensitive than strategies currently used in clinical practice. However, no single questionnaire or clinical model satisfies the criteria for the ideal preoperative screening test [42]. In the present study, the results of the administration of the Berlin questionnaire revealed a significant improvement between the preoperative and postoperative periods. The score prior to bariatric surgery demonstrated a high risk for OSA, whereas the results 90 days after surgery demonstrated a low risk. This finding was in accordance with the gold standard for the identification of OSA (PSG), for which a significant reduction of the postoperative AHI was found. To our knowledge, this is the first study where the Berlin questionnaire was administered to a population of patients with morbid obesity submitted to bariatric surgery.

The findings of this study demonstrate that weight loss following bariatric surgery led to a reduction of AHI and enhanced sleep architecture which is in agreement with data reported in previous studies that evaluated respiratory events and sleep architecture using preoperative and postoperative PSG [23,40,43]. Moreover, significant increases were found in the percentage of REM sleep and percentage of the deepest sleep stage N3.

A weakness of this and other studies in patients following bariatric surgery can be considered the relatively low proportion of patients who agreed to repeat the PSG following significant weight loss and the effect of the first night of the exam in the sleep laboratory, which can compromise sleep efficiency. Another limitation of the present study is the fact that the majority of patients were women, in whom a lesser impact on lung function is expected due to the distribution of fat in the female body.

## Conclusions

In conclusion, a high frequency of moderate-to-severe OSA was found in the initial sample of 52 patients planning bariatric surgery. The patients undergoing bariatric surgery met the criteria for OSA (75%) and 25% of these patients had moderate-to-severe OSA. The findings demonstrate that bariatric surgery for patients with severe obesity effectively reduces neck and waist circumference, improves pulmonary function, improves sleep architecture and reduces respiratory sleep disorders, especially OSA. The patients are currently in follow up for the determination of the results of bariatric surgery after one year.

## Competing interest

ICA, WRFJ, IRS, NA, SRN, JJU, NTF, FRT, EJI, PK, FSSLF, RMLN, CAM, GI, CFD, and LVFO declare that there is no association with any commercial enterprise that has interest in the object of this study. No competing of interest.

## Authors’ contributions

All the authors contributed to the conception and design the study. LVFO, ICA and WRFJ provided the idea of the study, established the hypothesis and wrote the original proposal. CAM, WRFJ, RMLN, FRT, EJI and PK performed the surgical procedures. IRS, SRN and NTF conducted the sleep studies. SRN and NA carried out pulmonary function tests. ICA, FSSLF, GI, CFD, FRT, EJI, PK and LVFO analyzed the data. This research paper was written by ICA, FSSLF, NA, CFD and LVFO, with input from all co-authors. IRS, CAM, WRFJ, RMLN, JJU, NTF, SRN, GI, and CFD were involved in critically revising the manuscript. All authors significantly contributed to this study and read and approved the final manuscript.
